# Severe Thrombocytopenia in a Patient With a History of Olfactory Neuroblastoma

**DOI:** 10.7759/cureus.33297

**Published:** 2023-01-03

**Authors:** Avani M Singh, Hailing Zhang, Lubomir Sokol

**Affiliations:** 1 Hematology and Medical Oncology, University of South Florida, Tampa, USA; 2 Hematology and Medical Oncology, Moffitt Cancer Center, Tampa, USA; 3 Pathology, Moffitt Cancer Center, Tampa, USA

**Keywords:** kadish, neuroendocrine, bone marrow, thrombocytopenia, olfactory neuroblastoma

## Abstract

Olfactory neuroblastoma is a rare disease with no randomized clinical trials to guide treatment decision making. Surgery, radiation, and chemotherapy are all used for treatment, and prognosis is mostly determined by the histologic grade and clinical stage. While a neuroendocrine type of neoplasm is similar to small cell carcinoma, metastatic disease in olfactory neuroblastoma is rare. We present a case at our institution of an uncommon clinical course of relapsed olfactory neuroblastoma complicated by severe thrombocytopenia.

## Introduction

Olfactory neuroblastoma (ONB), also known as esthesioneuroblastoma, is a rare malignant neoplasm that originates from the olfactory epithelium. Newly diagnosed ONB is a rare diagnosis itself, with an incidence of 0.4 in one million patients per year [1]. Arising from neural crest cells, this is a malignancy of the olfactory cells and is neuroendocrine in etiology. Presenting symptoms typically include nasal obstruction, anosmia, ear pain, diplopia, headache, or otitis media. Paraneoplastic syndromes, such as ectopic adrenocorticotropic hormone or syndrome of inappropriate antidiuretic hormone, can be rarely associated with this disease [2,3]. The vast majority is localized disease (Kadish stage A-C) managed with surgery, and metastatic disease (Kadish stage D) is rare with distant metastases in 8% of cases [4]. This is in contrast to neuroblastoma, where the disease presents with metastases in 50% of cases primarily involving bone marrow, bone, and vascular organs [5]. We present a unique case of this disease with rare associated complications not yet represented in the literature.

## Case presentation

A 55-year-old Caucasian woman with a history of high-grade locally advanced ONB presented for back pain and thrombocytopenia. She was diagnosed with ONB 15 months prior and had undergone treatment with concurrent radiation and chemotherapy with cisplatin and etoposide for three cycles. She achieved a complete response to treatment followed by surveillance with imaging every three months.

**Figure 1:**
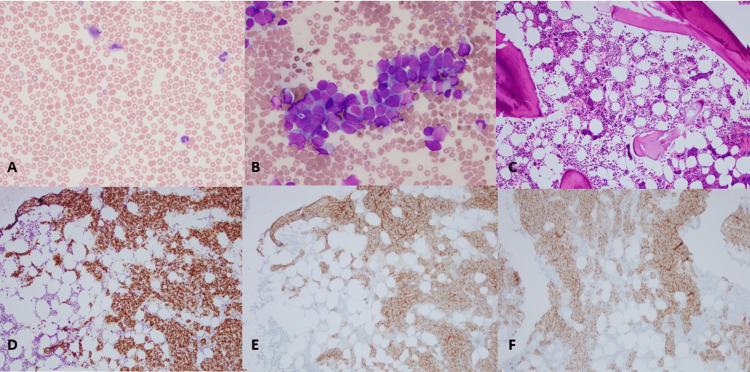
Peripheral smear and bone marrow evaluation A. Wright-stained peripheral blood smear showing marked thrombocytopenia. The neutrophils show mostly normal morphology. B. Wright-stained bone marrow aspirate smear with clusters of atypical large cells showing a high N/C ratio and fine chromatin. Mild to moderate nuclear polymorphism is present. C. Hematoxylin-eosin-stained bone marrow core biopsy shows clusters of neoplastic cell infiltrate. The residual trilineage hematopoiesis is seen here with an adequate number of megakaryocytes. The neoplastic cells seen on the bone marrow biopsy stained positive for INSM1 (D), SSTR2A (E), and synaptophysin (F) by immunohistochemistry.

On presentation, initial labs with a complete blood count (CBC) revealed a significantly low platelet count of 17.0 x 10^9^/L. The remainder of the CBC was normal including a hemoglobin (Hb) of 14.5g/dL, a mean corpuscular volume of 88.0, and a white blood cell count (WBC) of 10.7 x10^9^/L. The CBC differential showed an absolute neutrophil count (ANC) of 9.3 x 10^9^/L and a low absolute lymphocyte count of 0.54 x10^9^/L. Red blood cell morphology revealed 1+ polychromasia, poikilocytosis, and dacrocytes. The peripheral blood smear is depicted in Figure 1A. The patient’s baseline values were last known to be normal two months prior with a platelet count of 148.0 x10^9^/L, a Hb of 13.3 g/dL, a WBC of 4.0 x10^9^/L, and an ANC of 2.93 x 10^9^/L.

Hematology was consulted in the setting of isolated, severe thrombocytopenia. Initial investigation included normal coagulation studies, fibrinogen, immature platelet fraction of 8.6%, and peripheral smear with marked thrombocytopenia without platelet clumping or large platelets. Viral studies were negative, and no medications were contributing. While inpatient, her Hb began to downtrend daily by about 0.5g/dL. Hemolysis work-up including reticulocyte count, haptoglobin, and indirect bilirubin were normal. Bone marrow biopsy was performed, and Hb was 12.2g/dL at the time of the biopsy. The aspirate smear is seen in Figure 1B, and the core biopsy is in Figure 1C. Representative immunohistochemical (IHC) stains performed on the core biopsy are shown in Figure 1D-1F. The bone marrow biopsy demonstrated sheets of atypical cells with neuroendocrine features accounting for 70-80% of marrow space and reduced maturing trilineage hematopoiesis. By IHC staining, the atypical cells were positive for synaptophysin, calretinin, SSTR2A, and INSM1 consistent with extensive marrow involvement of metastatic ONB and negative for pan-keratin, chromogranin, and SOX10.

## Discussion

In the differential diagnosis for ONB are neuroendocrine carcinoma, melanoma, Ewing sarcoma, and sinonasal neuroendocrine neoplasm [6]. IHC for ONB is often positive for calretinin, CD56, chromogranin, synaptophysin, INSM1, and SSTR2, and some cells are positive for SOX10 [7]. To better understand the molecular profiling of advanced and metastatic ONB, Topcagic et al. performed NGS on 23 samples and results revealed common mutations in TP53, CTNNB1, EGFR, APC, cKIT, cMET, PDGFRA, CDH1, FH, and SMAD4 genes, no positive cases for PD-L1, and overexpression of pNTRK in 67% of cases [8]. A systematic review of the genomics of ONB by Kaur et al. revealed a similar complex genomic landscape [9]. Loss of 3p was the most common chromosomal alteration, with deletion of chromosome 11 and gain on chromosome 1p being associated with metastases and poor prognosis. TP53 was found to be the most frequently mutated gene, which is a frequently mutated gene contributing to tumorigenesis in multiple malignancies. 

In a retrospective study of 40 patients, all had localized disease, Kadish stage A-C, without any cases of metastatic disease [10]. Larger studies have found higher rates of metastases, though overall uncommon. In a systemic review examining 678 cases of the disease by Marinelli et al., 12% developed metastatic disease and were treated with systemic chemotherapy [7]. Most common sites of metastases included the bone, spine, and lungs, with median two-year overall survival of 63% after treatment with chemotherapy in addition to either surgery or radiation. In a 24-year experience at Istanbul University, 19 patients with ONB were treated. Twelve of the 19 ultimately developed metastases, and one of these included metastases to the bone marrow [11]. Bone marrow involvement is exceedingly rare but was present in a case report of ONB that presented with multiple bone metastases, which was observed by Zhou et al. [12].

The initial workup for thrombocytopenia in the present case revealed low concern for peripheral destruction or sequestration, but insufficient production of platelets was of the highest concern. Particularly in the setting of low IPF and significant dacrocytes on the peripheral blood smear, an infiltrative marrow process was a distinct possibility.

Secondary or therapy-related myeloid disorders, such as clonal cytopenia of undetermined significance (CCUS), myelodysplastic syndrome (MDS), and acute myeloid leukemia (AML), are most often seen in the first five years following exposure to chemotherapy and/or radiation therapy causing DNA damage. These cases are most common following treatment with alkylating agents, topoisomerase II inhibitors, or ionizing radiation. Cytogenetic testing often reveals complex karyotypes including deletion of 5q, deletion of 17p, or monosomies [13]. Molecular testing has shown that mutations in TP53, TET2, PTPN11, IDH1/2, and NRAS are often seen in therapy-related diseases [14]. This is a potential concern in the present case with prior therapy with etoposide. MDS is always important to consider in the setting of a single lineage cytopenia with prior chemotherapy treatment.

Immune thrombocytopenia (ITP) is always important to consider in the setting of isolated thrombocytopenia but is a diagnosis of exclusion. If alternatively, an overall normal bone marrow biopsy resulted, trialing empiric treatment for ITP would not be unreasonable. With declining hemoglobin during the hospitalization, thrombotic thrombocytopenic purpura (TTP) became important to exclude with negative hemolysis work-up and no schistocytes on peripheral blood smear. The development of concurrent hypoproliferative anemia made a secondary myeloid disorder or bone marrow infiltration a more likely diagnosis. While our patient’s thrombocytopenia was ultimately due to bone marrow infiltration by her malignancy, an initial broad differential must be included in the workup of acute severe thrombocytopenia.

## Conclusions

The patient was ultimately treated while in the hospital with a cycle of cisplatin and etoposide due to almost daily platelet transfusion requirements. Her course was complicated by neutropenic fever, mucositis, and sepsis due to multi-organism bacteremia. Due to a prolonged hospitalization, she decided to focus on comfort measures and was discharged to inpatient hospice care. As per our knowledge, this is the first case in the literature of recurrent metastatic olfactory neuroblastoma manifesting initially with only thrombocytopenia. This case illustrates the importance of maintaining a broad differential in approaching patient care, particularly in the setting of unusual diseases. Despite the rarity of metastatic disease in ONB, and even less common bone marrow involvement, this was the ultimate culprit of our patient’s acute thrombocytopenia.
